# Integrated Flexible Electronic Devices Based on Passive Alignment for Physiological Measurement

**DOI:** 10.3390/s17040889

**Published:** 2017-04-18

**Authors:** Jin Hwa Ryu, Sangwon Byun, In-Bok Baek, Bong Kuk Lee, Won Ick Jang, Eun-Hye Jang, Ah-Yung Kim, Han Yung Yu

**Affiliations:** 1 Bio-Medical IT Convergence Research Division, Electronics and Telecommunications Research Institute (ETRI), Daejeon 34129, Korea; gas96@etri.re.kr (J.H.R.); ibbaek@etri.re.kr (I.-B.B.); bklee32@etri.re.kr (B.K.L.); wijang@etri.re.kr (W.I.J.); cleta4u@etri.re.kr (E.-H.J.); aykim@etri.re.kr (A.-Y.K.); 2 Department of Electronics Engineering, Incheon National University, Incheon 22012, Korea; swbyun@inu.ac.kr

**Keywords:** flexible electronic device, passive alignment, electrocardiogram (ECG) sensor, interconnection

## Abstract

This study proposes a simple method of fabricating flexible electronic devices using a metal template for passive alignment between chip components and an interconnect layer, which enabled efficient alignment with high accuracy. An electrocardiogram (ECG) sensor was fabricated using 20 µm thick polyimide (PI) film as a flexible substrate to demonstrate the feasibility of the proposed method. The interconnect layer was fabricated by a two-step photolithography process and evaporation. After applying solder paste, the metal template was placed on top of the interconnect layer. The metal template had rectangular holes at the same position as the chip components on the interconnect layer. Rectangular hole sizes were designed to account for alignment tolerance of the chips. Passive alignment was performed by simply inserting the components in the holes of the template, which resulted in accurate alignment with positional tolerance of less than 10 µm based on the structural design, suggesting that our method can efficiently perform chip mounting with precision. Furthermore, a fabricated flexible ECG sensor was easily attachable to the curved skin surface and able to measure ECG signals from a human subject. These results suggest that the proposed method can be used to fabricate epidermal sensors, which are mounted on the skin to measure various physiological signals.

## 1. Introduction

Flexible electronics is a technology for integrating electrical components and a conductive interconnect layer on a flexible substrate, which has attracted much interest as an emerging field for next-generation electronics, such as lightweight, rugged, portable, bendable, or potentially foldable devices. To exploit these versatile applications, recent studies have focused on developing structure design strategies, fabrication techniques, and materials of flexible devices, which can replace conventional rigid devices used in the fields of biotechnology, communication systems, and display [1,2,3,4,5,6,7,8,9].

Manufacturing of functional electronic devices requires complex processes involving the fabrication of an interconnect layer which enables connections between chip components, and chip mounting on the interconnect layer. Previous studies on the interconnect layer have generally focused on improving stretchability, flexibility, and dimensional accuracy, using various fabrication technologies such as screen printing, inkjet, laser, soft lithography, and photolithography [10,11,12,13,14,15]. In addition, research on chip mounting has been conducted to increase positional alignment accuracy between the interconnect layer and chip components, since the alignment and bonding can significantly affect the reliability of fabrication. Chip mounting, also known as surface mount technology (SMT), is performed by first applying solder paste to the interconnect layer and then directly aligning each chip component. However, conventional SMT requires specific equipment for active alignment, including multiple precision stages, position controllers, and software for controllers, which significantly increase the cost. To overcome these limitations, existing literature has suggested self-alignment between the components and the interconnect layer. However, this may require a complicated fabrication process of three-dimensional structures [16,17]. Therefore, a simpler method of alignment is needed to perform efficient fabrication of functional electronic devices.

The aim of this study is to develop a fabrication technique for flexible devices using a simple method that enables passive alignment of chip components to the interconnect layer. To demonstrate feasibility, a flexible electrocardiogram (ECG) sensor was fabricated using the proposed method and tested on a human subject [18].

## 2. Concept of Passive Alignment for Fabrication of Functional Electronic Devices

**Figure 1:**
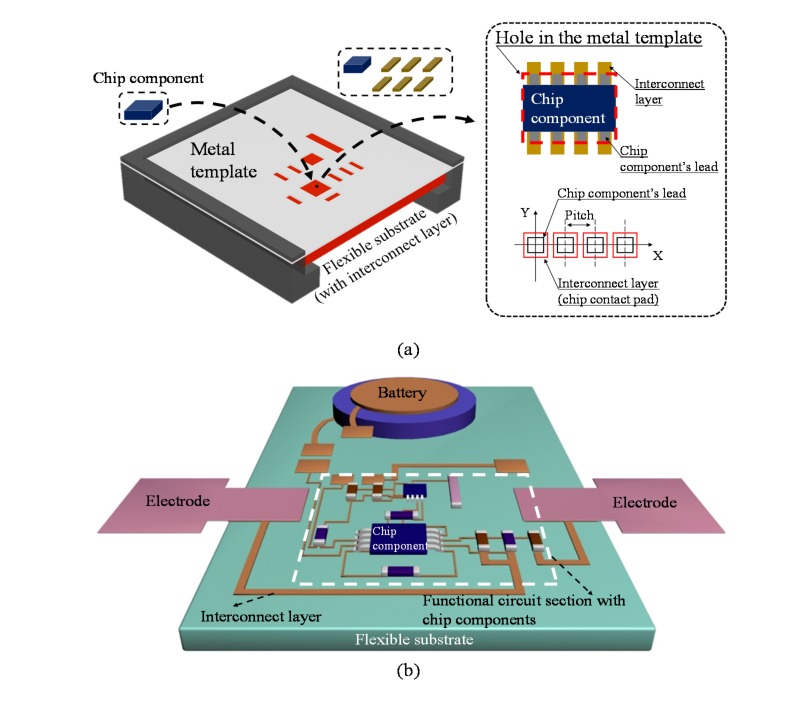
Schematic configurations of (**a**) passive alignment between chip components and an interconnect layer and (**b**) a flexible electronic sensor device.

In this study, we demonstrated a simple method to efficiently achieve accurate alignment between an interconnect layer and chip components, which significantly affects the fidelity of functional electronic devices (Figure 1). For example, misalignment between the interconnect layer and components, such as axial gap, lateral offset and angular misalignment, can cause loss of connection, crosstalk, or a short circuit. In particular, alignment accuracy of a small chip with narrow pitch is a critical factor for its performance. To address this problem, we fabricated a metal template with rectangular holes that guided the placement of the components (Figure 1a). The template had rectangular holes at the same position as the chip components on the interconnect layer. Rectangular hole sizes were designed to account for the alignment tolerance of the chips. For chip mounting, the metal template was placed on top of the interconnect layer built on the flexible substrate, and chip components were inserted in the holes. We used the self-alignment effect of solder paste to address any possible misalignment of the chips [1]. This made the metal leads of the mounted components align with the contact pads on the interconnect layer, where only a minimum margin of error was allowed for soldering connections. Therefore, passive alignment guided by the metal template can be used to facilitate accurate alignment between the interconnect layer and components [19].

**Figure 2:**
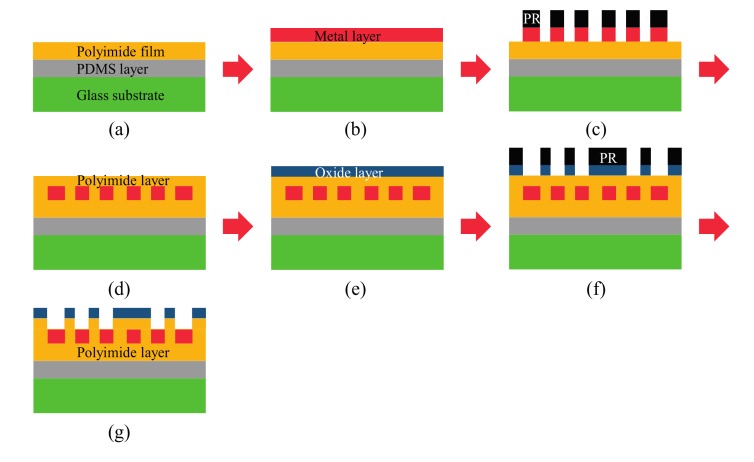
Schematic of the first part of the fabrication procedure: (**a**) deposition of the polydimethylsiloxane (PDMS)/polyimide (PI) film layers; (**b**) deposition of the metal layer (Ti/Cu) by E-beam evaporation; (**c**) patterning of the interconnect layer by the first photolithography; (**d**) deposition of the insulation layer by spin coating; (**e**) deposition of the SiO_2_ layer by plasma-enhanced chemical vapor deposition (PECVD); (**f**) patterning of the SiO_2_ layer by the second photolithography; (**g**) etching of the insulation layer to complete the fabrication of the sensor device platform.

**Figure 3:**
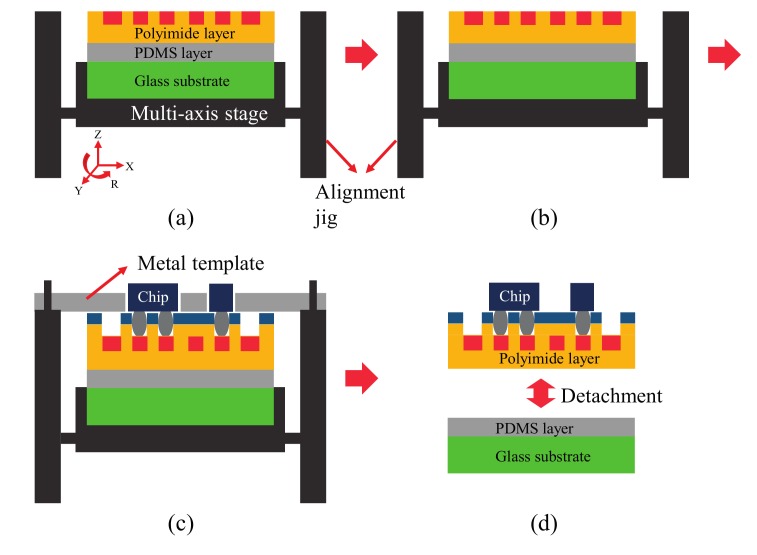
Schematic of the second part of the fabrication procedure: (**a**) alignment between the stencil mask and sensor device platform; (**b**) screen-printed solder paste on the interconnect layer; (**c**) passive alignment of chip components; (**d**) release of the flexible sensor device.

Figure 1b shows a schematic diagram of a flexible sensor device with two epidermal electrodes and a battery connected to the circuit. The device consisted of three structural layers: flexible substrate (electrically insulating), interconnect layer (conductive), and insulation layer (Figure 2 and Figure 3). The interconnect layer enabled electrical connections and signal transmission between chip components. The insulation layer covered the interconnect layer to prevent electrical interference and short circuit, except the areas for contact pads where chip components and epidermal electrodes were connected. A more detailed explanation of the structural layers can be found in the following section describing the fabrication procedures. The device was designed to operate as an ECG sensor to test the feasibility of the proposed method. Here, we used the circuit design suggested by Xu and colleagues, who successfully demonstrated a flexible ECG sensor operated by a small number of chip components on a flexible substrate [1]. We modified their circuit design to reduce the number of chip components (total 11 chips) while keeping key functions for ECG measurement, such as amplification and filtering. The spatial arrangement of chip components, contact pads, and a battery was optimized to reduce the complexity of interconnections. The white dashed box in Figure 1b indicates the functional circuit section, where passive alignment was applied to ensure reliable connections between the components and the interconnect layer as described above.

## 3. Fabrication of Flexible ECG Sensor Devices

A flexible sensor device was fabricated using two-step photolithography and passive alignment. As a flexible substrate, 20 µm thick polyimide (PI) film was used. Schematic illustrations of the fabrication processes are shown in Figure 2 and Figure 3. The overall procedure was divided into two parts: in the first part, a sensor device platform was built by forming an interconnect layer on the flexible substrate (Figure 2), and in the second part, the fabrication of a sensor device was completed by integrating chip components into the sensor device platform (Figure 3).

The first part of the fabrication procedure was based on the 4-inch glass wafer process, which was composed of two steps of photolithography for patterning the interconnect layer and insulation layer (Figure 2). The glass wafer was only used during the fabrication process as a carrier substrate to facilitate and stabilize the process. Therefore, the glass wafer was removed from the flexible device at the end of the process. First, a clean glass substrate was coated with a 100-µm thick layer of polydimethylsiloxane (PDMS, Sylgard 184, Dow Corning, Auburn, MI, USA). This PDMS layer was added onto the glass substrate to ensure separation of the flexible sensor device at the end of the fabrication procedure. The 20-µm thick PI film was physically attached to the surface of the PDMS layer as a flexible substrate using the backside adhesive of the PI film. The top surface of the PI film was treated with O_2_ plasma (O_2_ gas, 100 mL/min, 200 W, 60 s) to facilitate adhesion of the metal layer, which was formed by depositing Ti (5 nm) and Cu (400 nm) using E-beam evaporation. The first photolithography, which defined patterns of the interconnect layer, used a photoresist layer (PR, AZ 5214-E, 5000 rpm, 30 s) as an etch mask. Contact pads on the photomask were slightly oversized to include a 100-µm margin for chip dimensions. The Cu layer was etched by 0.5 M ammonium persulfate (Sigma Aldrich, St. Louis, MO, USA) for 90 s and the Ti layer by reactive ion etching (RIE) for 150 s using 20 sccm CF_4_ gas with 40 W RF power. To form an insulation layer, PI solution (poly(pyromellitic dianhydride-co-4,4′-oxydianiline), 5–12 poise, Sigma Aldrich) was spin-coated on the top surface, resulting in a 2.4-µm thick PI layer. A 50-nm thick SiO_2_ layer was deposited as an etch mask using plasma-enhanced chemical vapor deposition (PECVD). The second photolithography, which defined patterns of the insulation layer, used the same PR as the first photolithography. The SiO_2_ layer was etched by RIE for 10 min using 20 sccm CF_4_ and 6 sccm Ar gases with 20 W radio frequency (RF) power. The PI insulation layer was then etched by RIE for 30 min using 20 sccm O_2_ gas with 20 W RF power. Finally, the 50 mm × 50 mm sensor device platform was sawed from the wafer.

The second part of the procedure was composed of screen printing and chip mounting (Figure 3). First, a laser-cut stencil mask for screen printing was aligned with a sensor device platform. Alignment was precisely controlled by the custom-made jig with a manual stage, which provided XYZ translation and rotation around the *Z*-axis. The sensor device platform was placed on the manual stage. Positioning of the stencil mask, which was placed on top of the jig, was guided by matching the alignment holes of the mask and the alignment pillars of the jig. The Sn_42_Bi_58_ alloy solder paste (SMDLTFP250T3, Chip Quik, Niagara Falls, NY, USA) was then applied to the surface of the stencil mask to coat the contact pads on the interconnect layer with the paste. The stencil mask was removed and replaced with the metal template for chip mounting. Since the stencil mask and the metal template had the same size (width × length), and the same alignment holes as well, the manual stage was able to remain in the same position, which prevented any disruption of alignment. In particular, since the stencil mask and the metal template were fabricated by the same laser-cut process, alignment tolerance was less than 10 µm. Each chip component was then placed into its corresponding hole of the metal template. To ensure that chips can be easily inserted and to facilitate the removal of the metal template, the rectangular holes were slightly oversized to include a 50-µm margin for chip dimensions. After all chips were inserted, the metal template was carefully removed and the sensor device platform was placed in a vacuum oven for 5 min at 180 °C. Under this temperature, the surface tension of the solder paste caused motion that maximized the contact area to the contact pads on the interconnect layer, resulting in self-alignment of the components, which addressed any possible misalignment of the chips [1]. To release the flexible sensor device, the glass substrate and PDMS layer were removed from the backside, and this detachment was feasible due to the low surface energy of the PDMS layer and the flexibility of the sensor device.

## 4. Results and Discussion

**Figure 4:**
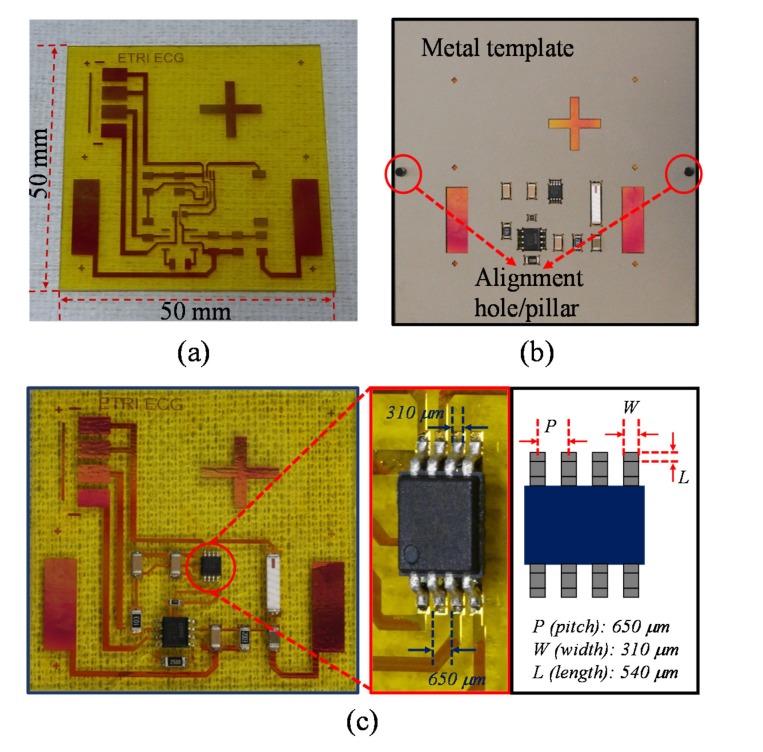
Optical images of products from various stages of the fabrication procedure: (**a**) a fabricated sensor device platform; (**b**) passive alignment of chip components on the sensor device platform using the metal template; (**c**) the complete sensor device. The magnified image shows the smallest active component on the circuit and its dimensions.

Products from various stages of the fabrication procedure are shown in Figure 4. The image of the sensor device platform clearly shows patterns of the interconnect layer formed by the first part of the fabrication (Figure 4a). It is important to note that the interconnect layer was mostly covered by the insulation layer, except the areas for contact pads where chip components and epidermal electrodes were connected. As described in Figure 2, these exposed areas for electrical connections were patterned by etching the insulation layer with RIE. Since the Cu layer could be affected by the RIE process, we measured changes in resistance and thickness of the Cu layer using the four-point probe (CMT-SR 2000 N, AIT, Suwon, Korea) and stylus-based surface profiler (Alpha-Step IQ, KLA Tencor, Milpitas, CA, USA), respectively. The change in resistance after RIE was less than 1 mΩ/sq and the change in thickness was negligible, suggesting that the RIE process did not affect the electrical characteristics of the Cu layer. Figure 4b shows a metal template placed on top of the sensor device platform with chip components inserted in the holes. The red-colored Cu layer underneath the metal template was partially visible through the unoccupied holes. Alignment between the metal template and the sensor device platform was guided by the alignment holes and pillars, which are indicated by red arrows in Figure 4b. Figure 4c shows the final product of the overall procedure. The magnified image of the smallest active component on the circuit shows accurate alignment with a positional tolerance of less than 10 µm with respect to the center-line of the contact pad on the interconnect layer and cleanly soldered connections without spill-over, demonstrating the ability of the metal template to efficiently align chip components with high accuracy.

**Figure 5:**
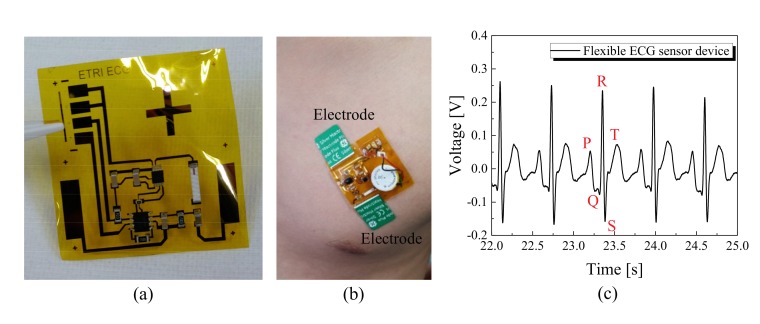
Images of the flexible sensor device and its operation as an electrocardiogram (ECG) sensor: (**a**) the flexible ECG sensor device detached from the PDMS layer/glass substrate; (**b**) the flexible sensor device attached to curved skin surface of chest; (**c**) ECG signals measured by the flexible sensor device.

In addition, we demonstrated that the fabricated flexible devices can be operated as a sensor for ECG measurement. To release a flexible sensor device, the glass substrate and PDMS layer were removed from the backside of the sensor device platform (Figure 5a). Since the device was built on a 20-µm thick PI film, it was easily bent during the separation, which might cause breakage of interconnect lines or detachment of mounted components. We examined the circuit of the flexible device with a microscope and a multi-meter to confirm the absence of defect connections. For power supply, a 3.7 V coin-type battery was connected to the circuit. The sensor device was easily attachable to the curved surface of the chest (Figure 5b). It could be laminated onto the skin surface using a 200-µm thick silicone-based film, which was attached to the backside of the device as a double-sided adhesive. The ECG signal from a human subject was measured in standard lead II configuration by placing two electrodes on the left pectoral region [20]. The output signal of the device was obtained by NI USB-6251 DAQ (National Instruments, Austin, TX, USA) and then processed by PC using Labview. To remove noise, a band-stop filter (Butterworth, second-order, 45–75 Hz) and a band-pass filter (Butterworth, third-order, 0.5–20 Hz) were used. Filtered output signals clearly showed typical features of lead II ECG waveform, including P-wave, QRS complex and T-wave, confirming that the circuit on the flexible substrate successfully operated as an ECG sensor (Figure 5c). These results suggested that our method can be used to fabricate non-invasive wearable sensors, which are mounted on the skin to measure physiological signals.

## 5. Conclusions

We have shown the value of using passive alignment for the fabrication of flexible electronic devices. The feasibility of our proposed method was tested by fabricating a flexible sensor device for ECG measurement. The sensor device was built on a 20-µm thick PI film with the dimensions of 50 mm × 50 mm, which successfully measured an ECG signal from a human subject. The device was also attachable to a curved skin surface, suggesting that the proposed method can be used to fabricate flexible device sensors for physiological signals. In this study, we operated passive alignment manually to verify the proof of concept. However, this method can be further improved by optimizing and standardizing the process, which may enable mass production, and also increase yield and reliability as high as those of SMT. Although we only presented a flexible device as an ECG sensor in this study, possible future applications include various types of wearable electronics and biomedical devices.
